# Seasonal Variations of Vogt-Koyanagi-Harada Disease in Japan: A Study on Long-Term Trends and the Influence of Coronavirus Disease 2019 (COVID-19)

**DOI:** 10.1016/j.xops.2025.100902

**Published:** 2025-07-31

**Authors:** Yuki Mizuki, Akira Meguro, Koji Yamamoto, Tatsukata Kawagoe, Nobuyuki Horita, Hiroyuki Okada, Norihiro Yamada, Takuto Sakono, Mami Ishihara, Shigeaki Ohno, Nobuhisa Mizuki

**Affiliations:** 1 Department of Ophthalmology and Visual Science, Yokohama City University Graduate School of Medicine, Yokohama, Kanagawa, Japan; 2 Department of Ophthalmology, Yokohama Minami Kyousai Hospital, Yokohama, Kanagawa, Japan; 3 Department of Biostatistics, Yokohama City University Graduate School of Medicine, Yokohama, Kanagawa, Japan; 4 Department of Ophthalmology, Dokkyo Medical University, Mibu, Tochigi, Japan; 5 Department of Pulmonology, Yokohama City University Graduate School of Medicine, Yokohama, Kanagawa, Japan; 6 Department of Ophthalmology, Okada Eye Clinic, Yokohama, Kanagawa, Japan; 7 Department of Ophthalmology, Faculty of Medicine and Graduate School of Medicine, Hokkaido University, Sapporo, Hokkaido, Japan

**Keywords:** Vogt-Koyanagi-Harada disease, Seasonality, COVID-19, Vaccination, Japan

## Abstract

**Objective:**

To investigate seasonal trends in the onset of Vogt-Koyanagi-Harada (VKH) disease in Japan and evaluate the influence of the coronavirus disease 2019 (COVID-19) pandemic, including severe cases and vaccination campaigns, on disease onset.

**Design:**

A retrospective cohort study using clinical records and public health data.

**Participants:**

A total of 320 Japanese VKH patients with a known month of onset who were initially treated at Yokohama City University Hospital between April 2007 and March 2024.

**Methods:**

The monthly distribution of VKH onset cases was assessed using the Roger test to determine seasonality before and after the COVID-19 pandemic. Severe COVID-19 case data (April 2020 to March 2024) were obtained from government sources. The COVID-19 vaccine administration data (April 2021 to March 2024) were collected from a major local clinic. Multiple linear regression was used to evaluate the temporal relationship between VKH onset and severe COVID-19 cases or vaccination, incorporating current-month (lag0), 1-month lag (lag1), and 2-month lag (lag2) predictors.

**Main Outcome Measures:**

Monthly number of VKH onset cases, seasonal trends, and statistical associations with COVID-19-related variables.

**Results:**

Seasonal variation was significant in VKH onset both before (*P* = 0.02) and after (*P* < 0.001) the pandemic, with a shift in peak onset from spring to late summer/fall. Vogt-Koyanagi-Harada onset was significantly associated with severe COVID-19 cases in the current month (β = 0.097; *P* = 0.02) and with COVID-19 vaccination counts with a 1-month delay (β = 0.83; *P* = 0.002). The regression models showed moderate to strong explanatory power (adjusted *R*^*2*^ = 0.52 and 0.80, respectively).

**Conclusions:**

This study revealed a postpandemic shift in the seasonal distribution of VKH onset in Japan, with peaks aligning with increased severe COVID-19 infections in the current month and vaccination activity in the preceding month. The findings suggest that both acute viral immune responses and delayed vaccine-induced immunity may contribute to VKH onset, highlighting the need for further investigation into the immunological mechanisms linking COVID-19 and autoimmune disease expression.

**Financial Disclosure(s):**

The authors have no proprietary or commercial interest in any materials discussed in this article.

Vogt-Koyanagi-Harada (VKH) disease is a multisystem autoimmune disorder that primarily affects melanocytes in the eyes, auditory system, and CNS.^1^^,^^2^ It is a major cause of endogenous uveitis in Japan, along with sarcoidosis and Behçet disease.^3^ Vogt-Koyanagi-Harada is more prevalent in East Asian populations,^4^ accounting for approximately 7% of uveitis cases in Japan,^3^ compared to 1% to 4% in the United States.^1^

The onset of VKH is strongly associated with human leukocyte antigen-DRB1∗0405, particularly in East Asians.^5^ Although genetic factors play a crucial role, the etiology of VKH and other autoimmune diseases remains complex and not fully understood. Environmental factors may also contribute to disease onset and progression.

Viral infections may act as environmental triggers, possibly via molecular mimicry involving cluster of differentiation 4–positive T cells.^6^ Viral infections, including hepatitis C and cytomegalovirus, have been suggested as possible triggers of VKH.^6^^,^^7^ Recently, several cases of VKH have been reported following coronavirus disease 2019 (COVID-19) infection or vaccination, typically occurring within several days to 1 month.^8^, ^9^, ^10^, ^11^ In addition to COVID-19 vaccines, VKH onset has also been reported following administration of other vaccines, including those against influenza and hepatitis B, suggesting that vaccine-induced immune activation may serve as a nonspecific environmental trigger in genetically predisposed individuals.^12^^,^^13^

Seasonal factors such as vitamin D, melatonin, and infectious agents have also been proposed as modulators of autoimmune activity.^14^ Because these factors vary by region, seasonal variations in autoimmune diseases may also differ geographically. To our knowledge, the first observation of seasonal variations in VKH was made by Ohno et al in 1988. Their analysis of a cohort of 186 Japanese patients showed a higher incidence of VKH onset in spring and late fall.^15^ In addition, a report summarizing data on uveitis patients in China from 2010 to 2019 showed that VKH onset peaked in February, with a smaller peak observed in May.^16^ Given the limited and dated nature of previous studies in Japan, a reassessment of VKH seasonality using recent data is warranted.

This study aims to evaluate current seasonal patterns of VKH onset in Japan and assess potential associations with COVID-19-related factors, including infection severity and vaccination.

## Methods

### Patient Selection and Diagnosis

The medical records of Japanese VKH patients with a documented month of onset were reviewed. All patients underwent initial evaluation and treatment for VKH at Yokohama City University Hospital between April 2007 and March 2024. All patients were diagnosed according to the Revised Diagnostic Criteria for VKH and met the criteria established at the first International Workshop on VKH Disease in 2001.^17^ A total of 320 patients met the inclusion criteria. Written informed consent was obtained. Patient data, including onset month, age, gender, and clinical features, were extracted from medical records. Vogt-Koyanagi-Harada onset was defined as the first appearance of ocular or prodromal symptoms. Clinical parameters included anterior uveitis, serous retinal detachment, and systemic symptoms such as tinnitus. Comparisons between pre- and post-pandemic cases were performed using the chi-square test, with odds ratios (ORs) and 95% confidence intervals (CIs). A *P* value <0.05 was considered statistically significant.

### Seasonal Variation in VKH Onset

To analyze seasonal variation in VKH onset from April 2007 to March 2024, the number of new cases per month was summed across all years to examine potential periodic trends. To statistically evaluate whether VKH onsets were uniformly distributed throughout the year, Roger test was applied, as it detects deviations from uniformity by using an efficient score vector to identify a single peak month (hereafter referred to as seasonality). Harmonic periodicity was evaluated by dividing a circle into 12 equal sectors and plotting monthly frequencies accordingly. The null hypothesis assumed no seasonality.^18^ To evaluate the impact of the COVID-19 pandemic, cases were divided into before March 2020 and from April 2020 onward. Furthermore, a 2 × 12 contingency table was constructed to compare monthly case distributions, and a chi-square test was applied.

### Seasonal Variation in Severe COVID-19 Cases

Roger test was also applied to the number of severe COVID-19 cases (April 2020–March 2024) to evaluate its seasonal variation. To reduce data duplication, the monthly average number of severe cases was used. Data were obtained from the Kanagawa Prefecture archive^19^ and the Ministry of Health, Labour and Welfare database.^20^

### COVID-19 Vaccination Data Collection

Data on monthly vaccine doses administered at Okada Eye Clinic (April 2021–March 2024) were collected. The clinic, a major vaccination center in Kanagawa Prefecture, administered approximately 50 000 doses annually.

### Comparison and Statistical Analysis

To evaluate the impact of severe COVID-19 cases and COVID-19 vaccination on VKH onset, a multiple linear regression analysis was conducted. The monthly number of VKH onset cases was used as the dependent variable, whereas the numbers of severe COVID-19 cases and COVID-19 vaccine doses were used as independent variables. To account for potential delayed effects, data for the current month (lag0), 1 month prior (lag1), and 2 months prior (lag2) were included as predictors. This model allowed for the evaluation of both immediate and time-dependent associations between VKH onset, severe COVID-19 cases, and vaccination counts.

### Statistical Tools and Compliance

Statistical analyses were performed using Python (version 3.9.7), including the Roger test, the chi-square test, and multiple linear regression analysis. Core Python libraries included NumPy, pandas, SciPy, and statsmodels. This study complied with the Declaration of Helsinki and was approved by the Institutional Review Board of Yokohama City University (protocol A110929003).

## Results

### Patient Characteristics

Among 320 VKH patients with known onset months, their demographic and clinical characteristics are summarized in Table 1. The median age at presentation was 52 years (range, 13–88), and all patients were East Asian residents of Japan. Women were slightly predominant (53.8%), and the proportion increased after the pandemic, from 49.3% before the pandemic to 64.5% after the pandemic (OR = 0.54 [95% CI: 0.33–0.88]; *P* = 0.02). Tinnitus was significantly more frequent before the COVID-19 pandemic (OR = 1.96 [95% CI: 1.07–3.69]; *P* = 0.02), as was sunset glow fundus (OR = 3.04 [95% CI: 1.45–7.01]; *P* = 0.002). In contrast, choroidal folds were more prevalent after the pandemic (OR = 0.48 [95% CI: 0.27–0.82]; *P* = 0.006). To further investigate this finding, we compared the prevalence of choroidal folds between patients diagnosed from April 2016 to March 2020 and from April 2020 to March 2024, which showed no significant difference (73.3% vs. 72.0%; *P* = 0.976).

**Table1:** Characteristics of Vogt-Koyanagi-Harada (VKH) Patients before and after the Coronavirus Disease 2019 (COVID-19) Pandemic

	All	Before the COVID-19 Epidemic	After the COVID-19 Epidemic	Odds Ratio	*P*
Number of patients (n)	320	227	93		
Median age (years)	52 (range: 13–88)	50 (range: 13–83)	56 (range: 24–88)		
Gender (%)					
Female	53.8	49.3	64.5	0.54 (0.33–0.88)	0.02
Male	46.2	50.7	35.5		
Race (%)					
East Asian descent	100.0	100.0	100.0		
Systemic complications (%)					
Tinnitus	29.7	33.5	20.4	1.96 (1.10–3.48)	0.03
Hearing loss	21.9	22.0	21.5	1.03 (0.57–1.85)	1.00
Vitiligo	1.9	2.2	1.1	2.07 (0.24–17.98)	0.82
Alopecia	1.6	1.3	2.2	0.61 (0.10–3.71)	0.96
Hypersensitivity to touch of hair and skin	12.2	10.1	17.2	0.54 (0.27–1.08)	0.12
Headache	55.9	53.7	61.3	0.73 (0.45–1.20)	0.27
Pyrexia	9.7	9.7	9.7	1.00 (0.44–2.27)	1.00
Vertigo	6.6	5.7	8.6	0.65 (0.26–1.61)	0.49
Nausea	3.8	3.1	5.4	0.56 (0.17–1.81)	0.51
Ocular findings (%)					
Active anterior uveitis	58.1	61.7	49.5	1.64 (1.01–2.67)	0.06
Exudative retinal detachment	90.0	89.9	90.3	0.95 (0.42–2.14)	1.00
Choroidal folds	60.0	55.1	72.0	0.48 (0.28–0.80)	0.007
Choroidal detachment	3.8	4.4	2.2	2.10 (0.45–9.76)	0.52
Optic disc edema	65.3	63.0	71.0	0.70 (0.40–1.17)	0.22
Sunset glow appearance of the fundus	22.2	26.9	10.8	3.04 (1.49–6.26)	0.003

COVID-19 = coronavirus disease 2019.

### Seasonal Variation of VKH Cases

**Figure 1:**
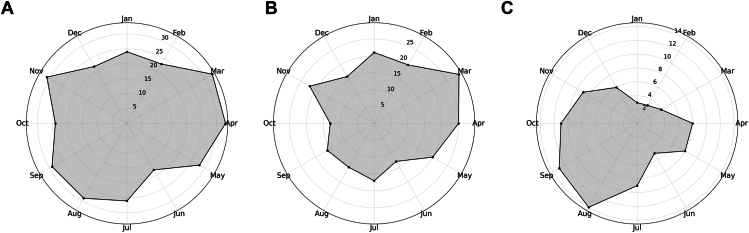
Seasonal variations of VKH onset cases. **A,** Monthly onset cases of VKH disease from April 2007 to March 2024. **B,** Vogt-Koyanagi-Harada onset cases before the COVID-19 pandemic (April 2007–March 2020). **C,** Vogt-Koyanagi-Harada onset cases after the COVID-19 pandemic (April 2020–March 2024). Each panel presents a polar plot of monthly case distribution. Shaded areas indicate case counts per month. COVID-19 = coronavirus disease 2019; VKH = Vogt-Koyanagi-Harada.

Figure 1A illustrates the monthly distribution of VKH onset cases by month from April 2007 to March 2024. The highest number of VKH cases was in March and April (33 cases each), followed by November (31 cases), whereas the lowest was in June (18 cases). Roger test showed no statistically significant seasonality (*P* = 0.93). Figure 1B illustrates the monthly distribution of VKH cases from April 2007 to March 2020, before the COVID-19 pandemic. The highest number of VKH cases was in March (29 cases), followed by April (25 cases) and November (22 cases). It appeared that VKH onset cases were more frequent in the spring. The lowest numbers were observed in June and October (13 cases each). Roger test indicated statistically significant seasonality (*P* = 0.02). To further examine prepandemic trends, we divided VKH patients diagnosed before the pandemic into 2 subgroups: April 2007–March 2016 and April 2016–March 2020. Roger test did not show statistically significant seasonality in either subgroup (*P* = 0.11 and *P* = 0.21, respectively). However, both distributions exhibited a consistent spring peak, particularly in March and April (Fig S1, available at www.ophthalmologyscience.org). Figure 1C illustrates the monthly distribution of VKH onset cases from April 2020 to March 2024, after the COVID-19 pandemic. The highest number of VKH cases was in August (14 cases), followed by September (13 cases) and October (11 cases). It appeared that VKH cases were more frequent from summer to fall. The lowest numbers were observed in January and February (3 cases each). Roger test indicated statistically significant seasonality (*P* < 0.001).

To compare the monthly distribution of VKH onset cases between the 2 groups before and after the COVID-19 pandemic, a 2 × 12 contingency table was created. The analysis revealed a statistically significant difference in the monthly distribution of VKH onset cases between the 2 periods (*P* = 0.015).

### Seasonal Variation of Severe COVID-19 Cases

**Figure 2:**
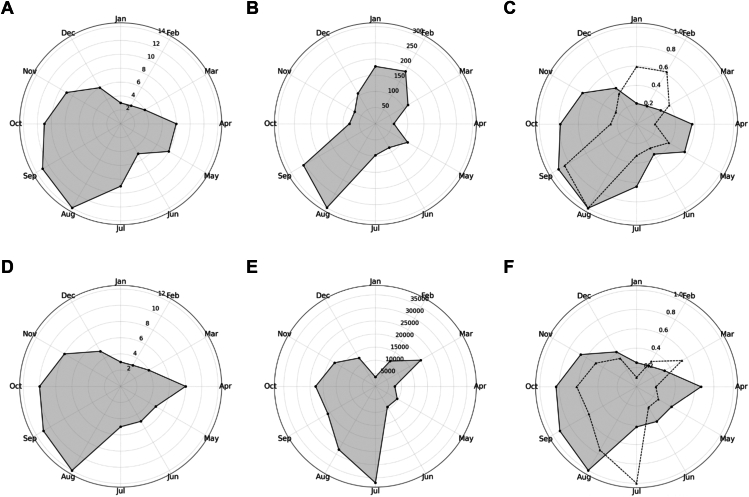
Seasonal variations in VKH cases, severe COVID-19 cases, and vaccination counts. **A,** Monthly onset cases of VKH disease (April 2020–March 2024). **B,** Monthly counts of severe COVID-19 cases (April 2020–March 2024). **C,** Scaled comparison of VKH onset (solid line) and severe COVID-19 cases (dotted line). **D,** Monthly VKH onset cases (April 2021–March 2024). **E,** Monthly COVID-19 vaccination doses administered (April 2021–March 2024). **F,** Scaled comparison of VKH onset (solid line) and COVID-19 vaccination counts (dotted line). Data in **(C)** and **(F)** are scaled to their respective maxima to facilitate comparison. COVID-19 = coronavirus disease 2019; VKH = Vogt-Koyanagi-Harada.

Additionally, we analyzed the seasonal variation of the number of severe COVID-19 cases from April 2020 to March 2024 (Fig 2B). The highest number of severe COVID-19 cases was observed in August (299.6 cases), followed by September (256.5 cases) and February (186.6 cases). The lowest numbers were observed in April (56.8 cases) and November (73.8 cases). Roger test confirmed a highly significant seasonality (*P* < 0.001).

To visualize the relationship between VKH onset and severe COVID-19 cases, a scaled polar plot was generated (Fig 2C). Both datasets were scaled to their maximum values for direct comparison. Vogt-Koyanagi-Harada cases and severe COVID-19 cases both showed peaks in late summer (August to September), suggesting a potential association.

### Seasonal Variation of VKH Onset and COVID-19 Vaccination Counts

The monthly distribution of VKH onset cases and COVID-19 vaccine doses administered from April 2021 to March 2024 were analyzed for seasonal patterns. Roger test indicated a statistically significant seasonality in VKH onset cases (*P* = 0.009). The highest number of VKH cases was observed in August (12 cases) and September (11 cases), suggesting a late-summer peak (Fig 2D). In contrast, the monthly variation in COVID-19 vaccine doses also showed a highly significant seasonality (*P* < 0.001), with peaks in July (36 945 doses) and August (27 994 doses) (Fig 2E). To visualize the relationship between VKH onset and COVID-19 vaccination counts, a scaled polar plot was generated (Fig 2F). The COVID-19 vaccination counts peaked in July, whereas VKH onset cases peaked in August, showing a 1-month delay between the 2 peaks.

### Multiple Linear Regression Analysis

To evaluate the effects of severe COVID-19 cases and COVID-19 vaccination on VKH onset, we performed multiple linear regression analyses. Each model included the current month's data (lag0), along with data from 1 month prior (lag1) and 2 months prior (lag2), as independent variables. The model fit, overall significance, and regression coefficients for each model are summarized in Table 2.

**Table 2:** Summary of Multiple Linear Regression Analysis for VKH Onset

Summary of Multiple Linear Regression Models					
Model	Dependent Variable	*R*^*2*^	Adjusted *R*^*2*^	F-Statistic	*P* Value
Severe COVID-19 cases	VKH onset	0.68	0.52	4.25	0.06
COVID-19 vaccination	VKH onset	0.87	0.80	13.20	0.005

Regression coefficients for each model			
Model	Variable	β (Standardized)	*P* Value
Severe COVID-19 cases vs. VKH cases	Lag0	0.097	0.02
	Lag1	−0.0095	0.74
	Lag2	0.55	0.11
COVID-19 vaccination vs. VKH cases	Lag0	0.079	0.63
	Lag1	0.83	0.002
	Lag2	0.19	0.27

Adjusted R² = adjusted coefficient of determination; β = standardized regression coefficient; COVID-19 = coronavirus disease 2019; Lag0 = current month; Lag1 = 1 month prior; Lag2 = 2 months prior; *P* value = probability value; R² = coefficient of determination; VKH = Vogt-Koyanagi-Harada.

**Figure 3:**
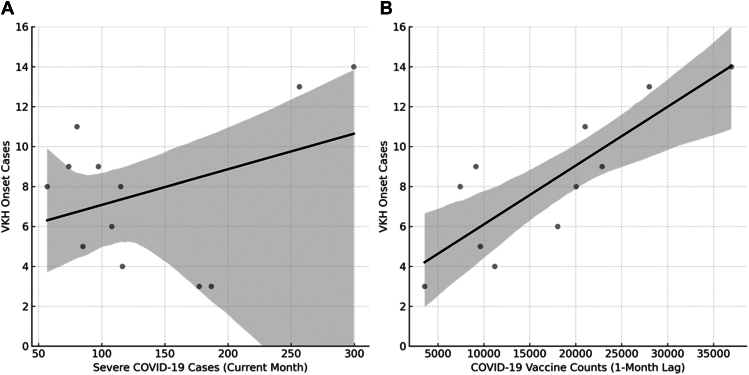
Associations between severe COVID-19 cases, vaccination, and VKH onset. This figure illustrates the relationships between severe COVID-19 cases, vaccination, and VKH onset based on multiple linear regression analysis. **A,** Association between severe COVID-19 cases in the current month (lag 0) and VKH onset. **B,** Association between COVID-19 vaccine doses administered in the prior month (lag 1) and VKH onset. Gray dots represent observed VKH onset cases, solid black lines indicate fitted regression models, and shaded areas denote 95% confidence intervals. COVID-19 = coronavirus disease 2019; VKH = Vogt-Koyanagi-Harada.

The results indicate that severe COVID-19 cases have an immediate effect on VKH onset (β = 0.97; *P* = 0.02), whereas COVID-19 vaccination influences VKH onset with a 1-month delay (β = 0.83; *P* = 0.002). No significant associations were observed for the lag1 and lag2 COVID-19 cases (lag1: *P* = 0.74; lag2: *P* = 0.11) or for lag0 and lag2 vaccination counts (lag0: *P* = 0.63; lag2: *P* = 0.27) (Table 2). Figure 3 illustrates these relationships, highlighting the significant associations.

## Discussion

### Demographic and Clinical Changes in VKH Patients

In this study, 53.8% of VKH patients were female, with a significant increase observed after the pandemic (from 49.3% before to 64.5% after; *P* = 0.02). Gender differences in immune and autoimmune responses are well recognized: women tend to have milder infections but more frequent vaccine-related adverse events,^21^^,^^22^ possibly contributing to this increase.

Systemic and ocular symptoms also differed between periods. Tinnitus was significantly more prevalent before the pandemic (*P* = 0.02), potentially reflecting changes in immune regulation or environmental factors, such as reduced stress and noise exposure during the pandemic. Among ocular symptoms, sunset glow fundus (*P* = 0.002) was more prevalent before the pandemic, likely reflecting shorter disease duration after the pandemic. Conversely, choroidal folds were more prevalent after the pandemic (*P* = 0.006), likely due to advances in OCT technology. A subanalysis (2016–2020 vs. 2020–2024) showed no significant difference (*P* = 0.976), supporting this explanation.

### Comparison of VKH Seasonality: Current Findings and Previous Reports

In this study, we evaluated the seasonality of VKH onset in Japan using the Roger test. Although no significant seasonal variation was observed among patients from April 2007 to March 2024 overall, separate analyses revealed seasonal differences before and after the COVID-19 pandemic. Specifically, VKH onset was more frequent in spring before the pandemic and peaked from August to October after. Subgroup analysis of 2007 to 2016 and 2016 to 2020 showed visual spring peaks despite no significant seasonality, supporting a prepandemic spring trend. Ohno et al, based on cases from 1966 to 1983 in Japan, reported VKH onset peaks in spring and late fall,^15^ and Hao et al, analyzing cases from 2010 to 2019 in China, noted peaks in February and May.^16^ These findings may be similar to the seasonal trends in VKH onset observed between April 2007 and March 2020 in our study. In contrast, a report from Puerto Rico, based on cases from 1986 to 2018, found higher incidence in the fall.^23^ However, because Puerto Rico has a tropical climate, direct comparison with Japan's seasonal patterns warrants caution.

### Seasonal Influence of Vitamin D Deficiency and Infectious Pathogen Exposure on Pre-COVID-19 VKH Onset

Vitamin D levels vary seasonally with ultraviolet exposure, and serum 25-hydroxyvitamin D (25[OH]D) levels are typically lowest in spring because of a one-season lag in ultraviolet-induced synthesis.^24^ Reduced vitamin D, acting as an immunomodulator, has been linked to increased autoimmune disease activity in spring, including multiple sclerosis and systemic lupus erythematosus.^14^^,^^25^ Similarly, the higher VKH onset observed in spring before the pandemic may be partly influenced by these seasonal fluctuations.

Infectious pathogen exposure also increases in winter to early spring in Japan, with group A streptococcal infections and influenza peaking during this period.^26^^,^^27^ This seasonal alignment suggests that both vitamin D deficiency and infection-driven immune activation may have contributed to the spring peak in VKH onset before the COVID-19 pandemic.

### Seasonal Variation of VKH Onset and Severe COVID-19 Cases

In our study, a statistically significant seasonal variation was observed in severe COVID-19 cases from April 2020 to March 2024, peaking in August (299.6 cases) and September (256.5 cases). Vogt-Koyanagi-Harada onset cases also peaked in August (12 cases) and September (11 cases), suggesting a possible temporal association. Multiple linear regression analysis revealed a significant association between VKH onset and severe COVID-19 cases in the current month (*P* = 0.02).

The regression model demonstrated moderate explanatory power (*R*^*2*^ = 0.68; adjusted *R*^*2*^ = 0.52). Although the overall model was marginally nonsignificant (F = 4.25, *P* = 0.06), this may reflect limited power because of the fixed 12-month dataset rather than absence of association. The detection of a significant predictor despite this limitation suggests that severe COVID-19 cases may contribute to VKH onset in the current month. These findings underscore the value of multiple regression in identifying associations not evident in unadjusted analyses.

### Association Between VKH Onset and COVID-19 Vaccination

The analysis of VKH onset and COVID-19 vaccination counts revealed a temporal association, with the effect appearing after a 1-month lag. The highest number of vaccine doses was administered in July (36 945 doses), followed by August (27 994 doses), whereas VKH onset peaked in August and September.

This relationship was supported by multiple linear regression, which identified 1-month lagged vaccination counts as a significant predictor (*P* = 0.002). The model demonstrated strong explanatory power (*R*^*2*^ = 0.87; adjusted *R*^*2*^ = 0.80), with an F-statistic of 13.20 (*P* = 0.004), confirming the robustness of the association. Although N was limited to 12, the strength and consistency of the findings support a delayed effect of vaccination on VKH onset.

### Mechanisms Linking COVID-19 Infection and Vaccination to VKH Onset

One potential mechanism by which COVID-19 infection may trigger VKH is the excessive activation of the immune system through a cytokine storm. During COVID-19 infection, levels of interferon-γ, interleukin)-6, and interleukin-17 are elevated, leading to increased T-cell activation.^6^ This may result in an autoimmune attack targeting melanocytes, ultimately leading to VKH onset. Notably, Santamaria et al have reported several cases of VKH after COVID-19 infection, suggesting a potential immunological trigger.^8^ Furthermore, molecular mimicry may also play a role, as the severe acute respiratory syndrome coronavirus 2 spike protein shares structural similarities with melanocyte antigens, such as tyrosinase, potentially triggering cross-reactive immune responses.^6^ These findings suggest that acute immune activation after COVID-19 infection may contribute to the immediate onset of VKH.

In contrast, VKH onset following COVID-19 vaccination appears to be mediated by a delayed immune response. mRNA vaccines strongly activate T-cell responses via lipid nanoparticles,^11^ with peak immune activation reported to peak approximately 1 to 2 weeks after vaccination in a recent case study.^28^ This delayed immune activation may enhance autoimmune responses, leading to VKH development. Additionally, vaccine adjuvants may further stimulate the immune system, amplifying inflammatory responses.^12^ The 1-month lag observed in our study suggests a delayed immune-mediated mechanism, which differs from previous case reports that primarily describe VKH onset occurring within days to weeks after vaccination.^9^^,^^10^^,^^29^ Although VKH recurrence after vaccination has been reported with a delayed onset,^30^ our findings indicate that even in new-onset VKH, a delayed response may be relevant at the population level. This suggests that vaccine-induced immune activation may continue over several weeks, potentially contributing to VKH onset 1 month after vaccination.

This study focused primarily on evaluating the seasonal patterns of VKH onset using population-level data. Although our findings suggest temporal associations with community-level trends in severe COVID-19 cases and vaccination counts, individual-level data on infection or vaccination status were not assessed in this cohort. A separate, ongoing study is currently investigating the temporal relationship between VKH onset and personal histories of COVID-19 infection or vaccination, including the detailed analysis of individual cases.

In conclusion, our findings demonstrate a postpandemic seasonal shift in VKH onset in Japan, with temporal peaks associated with severe COVID-19 cases in the current month and vaccination activity during the preceding month. Multiple linear regression analyses revealed a significant immediate association with severe COVID-19 infection and a delayed association with vaccination, suggesting that both acute viral immune responses and vaccine-induced immunity may contribute to VKH onset. These results underscore the influence of population-level immunologic shifts on autoimmune disease expression and warrant continued surveillance and mechanistic investigations.

## References

[1] Damico F.M., Kiss S., Young L.H. (2005). Vogt-Koyanagi-Harada disease. Semin Ophthalmol.

[2] Sakamoto T., Murata T., Inomata H. (1991). Class II major histocompatibility complex on melanocytes of vogt-koyanagi-harada disease. Arch Ophthalmol.

[3] Ohguro N., Sonoda K.H., Takeuchi M. (2012). The 2009 prospective multi-center epidemiologic survey of uveitis in Japan. Jpn J Ophthalmol.

[4] Moorthy R.S., Inomata H., Rao N.A. (1995). Vogt-Koyanagi-Harada syndrome. Surv Ophthalmol.

[5] Shindo Y., Inoko H., Yamamoto T., Ohno S. (1994). HLA-DRB1 typing of vogt-koyanagi-harada's disease by PCR-RFLP and the strong association with DRB1∗0405 and DRB1∗0410. Br J Ophthalmol.

[6] Sugita S., Takase H., Kawaguchi T. (2007). Cross-reaction between tyrosinase peptides and cytomegalovirus antigen by T cells from patients with vogt-koyanagi-harada disease. Int Ophthalmol.

[7] Touitou V., Bodaghi B., Cassoux N. (2005). Vogt-Koyanagi-Harada disease in patients with chronic hepatitis C. Am J Ophthalmol.

[8] Santamaria A., Chang J., Savarain C. (2022). SARS-CoV-2 among the potential viral triggers for vogt-konayagi-harada disease: first case report and literature review. Ocul Immunol Inflamm.

[9] Saraceno J.J.F., Souza G.M., Dos Santos Finamor L.P. (2021). Vogt-Koyanagi-Harada Syndrome following COVID-19 and ChAdOx1 nCoV-19 (AZD1222) vaccine. Int J Retina Vitreous.

[10] Yepez J.B., Murati F.A., Petitto M. (2021). Vogt-Koyanagi-Harada disease following COVID-19 infection. Case Rep Ophthalmol.

[11] Joo C.W., Kim Y.K., Park S.P. (2022). Vogt-Koyanagi-Harada Disease following mRNA-1273 (Moderna) COVID-19 vaccination. Ocul Immunol Inflamm.

[12] Sood A.B., O'Keefe G., Bui D., Jain N. (2019). Vogt-Koyanagi-Harada disease associated with hepatitis B vaccination. Ocul Immunol Inflamm.

[13] Murtaza F., Pereira A., Mandelcorn M.S., Kaplan A.J. (2022). Vogt-Koyanagi-Harada disease following influenza vaccination. Am J Ophthalmol Case Rep.

[14] Watad A., Azrielant S., Bragazzi N.L. (2017). Seasonality and autoimmune diseases: the contribution of the four seasons to the mosaic of autoimmunity. J Autoimmun.

[15] Ohno S., Minakawa R., Matsuda H. (1988). Clinical studies of vogt-koyanagi-harada's disease. Jpn J Ophthalmol.

[16] Hao T., Yang L.I., Li B. (2021). Epidemiology of 2000 Chinese uveitis patients from Northeast China. Br J Ophthalmol.

[17] Read R.W., Holland G.N., Rao N.A. (2001). Revised diagnostic criteria for vogt-koyanagi-harada disease: report of an international committee on nomenclature. Am J Ophthalmol.

[18] Roger J.H. (1977). A significance test for cyclic trends in incidence data. Biometrika.

[19] Kanagawa Prefecture New Coronavirus data archive. https://www.pref.kanagawa.jp/docs/ga4/covid19/archive/data.html?mode=preview.

[20] Ministry of Health, Labour and Welfare (Japan) COVID-19 open data. https://www.mhlw.go.jp/stf/covid-19/open-data.html.

[21] Oertelt-Prigione S. (2012). The influence of sex and gender on the immune response. Autoimmun Rev.

[22] Harris T., Nair J., Fediurek J., Deeks S.L. (2017). Assessment of sex-specific differences in adverse events following immunization reporting in Ontario, 2012-15. Vaccine.

[23] Amaral C., Rodriguez E., Barquet V. (2023). Seasonal patterns of vogt-koyanagi-harada disease. Ocul Immunol Inflamm.

[24] Maeda S.S., Saraiva G.L., Hayashi L.F. (2013). Seasonal variation in the serum 25-hydroxyvitamin D levels of young and elderly active and inactive adults in Sao Paulo, Brazil: the Sao PAulo Vitamin D Evaluation Study (SPADES). Dermatoendocrinol.

[25] Rosen Y., Daich J., Soliman I. (2016). Vitamin D and autoimmunity. Scand J Rheumatol.

[26] National Institute of Infectious Diseases (NIID), Japan (2015). Streptococcal Infections in Japan, 2012–2015, as of June 2015. Infect Agents Surveill Rep (Iasr).

[27] Shimmei K., Nakamura T., Ng C.F. (2020). Association between seasonal influenza and absolute humidity: time-series analysis with daily surveillance data in Japan. Sci Rep.

[28] Hirano H., Asada H. (2024). Exponential decline, ceiling effect, downregulation, and T-cell response in immunoglobulin G antibody levels after messenger RNA vaccine boosters: a case report. J Med Case Rep.

[29] Xu K., Gao B., Li J. (2023). Clinical features, diagnosis, and management of COVID-19 vaccine-associated vogt-koyanagi-harada disease. Hum Vaccin Immunother.

[30] Papasavvas I., Herbort C.P. (2021). Reactivation of vogt-koyanagi-harada disease under control for more than 6 years, following anti-SARS-CoV-2 vaccination. J Ophthalmic Inflamm Infect.

