# Supplementary material for: Seasonal Variations of Vogt-Koyanagi-Harada Disease in Japan: A Study on Long-Term Trends and the Influence of Coronavirus Disease 2019 (COVID-19)
Source: Ophthalmol Sci. 2025 Jul 31;6(1):100902. doi: 10.1016/j.xops.2025.100902 (PMC12494803; doi:10.1016/j.xops.2025.100902)
Supplement: Figure S1 [file mmc1.pdf]

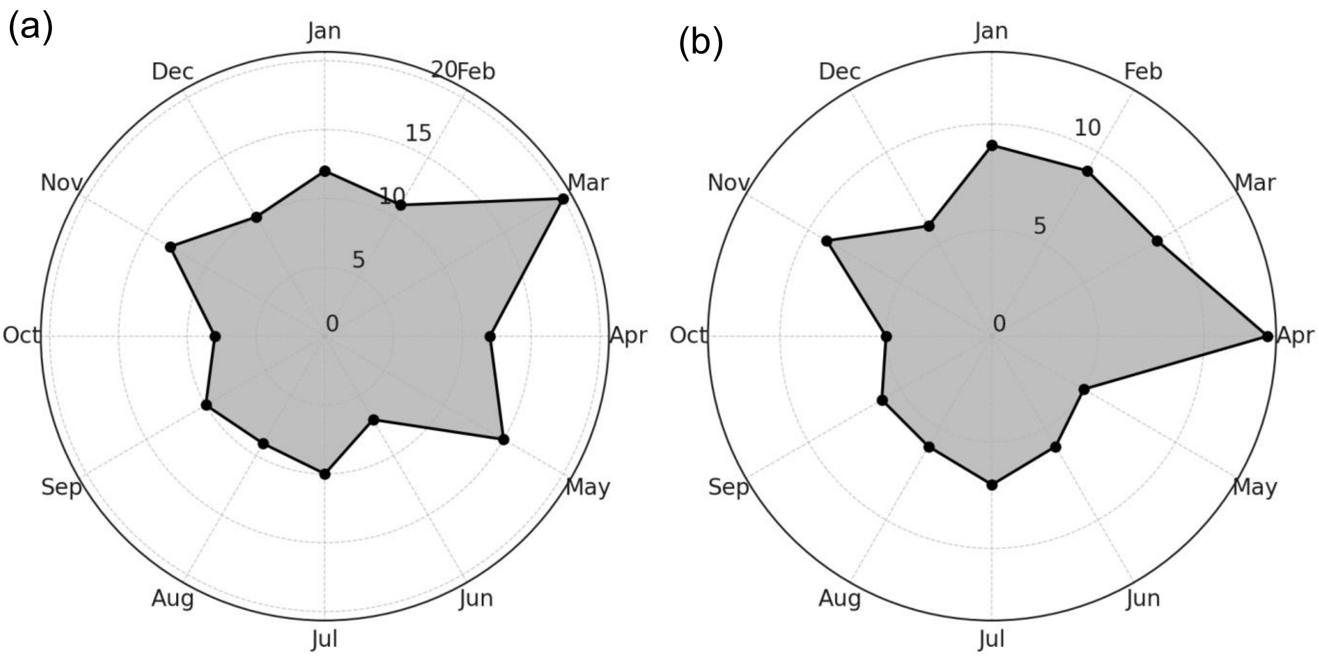

Supplemental Figure 1. Monthly VKH Onset Trends in Pre-Pandemic Subgroups

Monthly distribution of VKH onset cases before the COVID-19 pandemic. (a): 2007–2016; (b): 2016–2020. Although Roger’s test did not reveal statistically significant seasonality in either subgroup ($p = 0.11$ and $p = 0.21$, respectively), both distributions showed a consistent visual trend of increased VKH onset in the spring, particularly in March and April.
